# The Receiver of the *Agrobacterium tumefaciens* VirA Histidine Kinase Forms a Stable Interaction with VirG to Activate Virulence Gene Expression

**DOI:** 10.3389/fmicb.2015.01546

**Published:** 2016-01-08

**Authors:** Arlene A. Wise, Andrew N. Binns

**Affiliations:** Binns Lab, Department of Biology, University of Pennsylvania, PhiladelphiaPA, USA

**Keywords:** hybrid histidine kinase, response regulator, receiver domain, signal transduction, *Agrobacterium*, plant pathogenesis

## Abstract

The plant pathogen *Agrobacterium tumefaciens* carries a virulence gene system that is required for the initiation of crown gall tumors on susceptible plants. Expression of the *vir* genes is activated by the VirA/VirG two component regulatory system. VirA is a histidine kinase which signals the presence of certain chemicals found at the site of a plant wound. The receiver domain located at its carboxyl terminus defines VirA as a hybrid histidine kinase. Here, we show that the VirA receiver interacts with the DNA-binding domain of VirG. This finding supports the hypothesis that the receiver acts as a recruiting factor for VirG. In addition, we show that removal of the VirA receiver allowed *vir* gene expression in response to glucose in a dose dependent manner, indicating that the receiver controls VirG activation and suggesting that the supplementary ChvE-sugar signal increases this activity.

## Introduction

**FIGURE 1:**
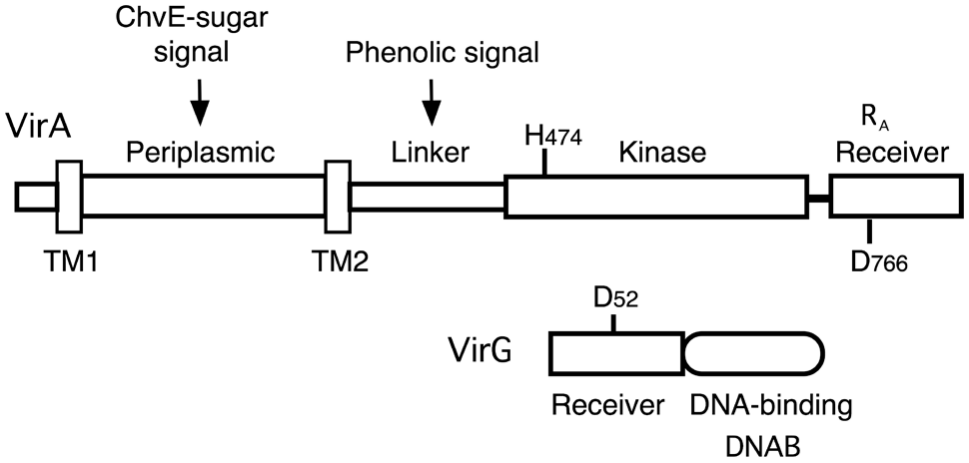
**Diagram of VirA and VirG.** VirA and VirG are arranged in functional domains. Key amino acids are indicated. H474 is the site of autophosphorylation in the VirA kinase domain. D52 is the aspartate in the VirG receiver domain that accepts the phosphate from VirA. D766N in the VirA receiver domain is analogous to VirG’s D52. R_A_ denotes the receiver domain of VirA and DNAB represents the DNA-binding domain of VirG.

*Agrobacterium tumefaciens* is the causative agent of crown gall tumors on dicotyledonous plants. The virulence system that drives tumor formation depends on the ability of the VirA histidine kinase to signal the presence of certain chemical components found at the site of a plant wound. When the inducing signals (phenolics, monosaccharides, and low pH) are present, a phosphate group is transferred from the conserved histidine (H474) in VirA’s kinase region to a conserved aspartate (D52) in the receiver domain of the response regulator, VirG (see **Figure 1**). Phosphorylated VirG is able to bind the promoters of the virulence (*vir*) genes and activate their transcription (for reviews, see Gelvin, 2003; McCullen and Binns, 2006).

The phenolic signal and slightly acidic pH are essential for *vir* gene induction. Certain monosaccharides (arabinose, glucose, galactose, glucuronic acid, and others), are not, by themselves, inducing agents. However, their presence greatly enhances *vir* gene expression when the concentration of phenolic inducer is low (Ankenbauer and Nester, 1990; Cangelosi et al., 1990). Reception of the phenolic signal requires the linker region of VirA (Chang and Winans, 1992) and likely involves a region that protein modeling indicates has structural similarity to a GAF domain (Gao and Lynn, 2007; Lin et al., 2014). The effect of sugar on the virulence system requires the periplasmic ChvE sugar-binding protein and is mediated via interaction of ChvE with the VirA periplasmic domain (Cangelosi et al., 1990; Chang and Winans, 1992; Shimoda et al., 1993; Hu et al., 2013).

VirA can be classified as a hybrid kinase because it carries a region with sequence similarity to the receiver domains of response regulators, including an aspartate at codon 766 that is analogous to the phosphorylatable aspartate (residue 52) in VirG’s receiver domain. We have previously suggested that VirA’s receiver domain functions differently than the receiver domains of hybrid kinases that are known to function as part of a phospho-relay (Wise et al., 2010). For example, while mutation of D766 reduces *vir* gene expression, it does not create a null mutation (Pazour et al., 1991). Furthermore, VirA does not include an HPT (histidine phospho-transfer) domain and *in vitro* experiments using purified proteins indicate that an HPT domain is not required for phosphate transfer between VirA and VirG (Jin et al., 1990). Most importantly, the VirA receiver is not essential for *vir* gene expression. Deletion of the receiver domain does change *vir* gene expression, but that effect varies significantly depending on how *virG* or *virA*ΔR are expressed. As shown in earlier studies that used constitutively expressed *virG*, VirAΔR is capable of activating *vir* gene expression in the absence of the normally essential phenolic inducer, provided glucose or arabinose is available (Chang and Winans, 1992; Chang et al., 1996). These early results defined the receiver as an inhibitor of *vir* gene expression. However, replacing wild type *virA* on the tumor-inducing plasmid (pTi) with *virAΔR* demonstrated that the receiver domain of VirA is essential for *vir* gene expression if wild type *virG* is expressed solely from its natural position on pTi (Wise et al., 2010). Thus, the receiver has both activating and inhibitory effects on *vir* gene expression.

In this study, we examined the positive and negative roles of the VirA receiver (R_A_) in regulating *vir* gene expression and found the following: (1) R_A_, expressed *in trans*, did not correct the null phenotype of a *virAΔR* strain. In fact, R_A_ inhibited *vir* gene expression in the wild type background more strongly than a mutant receiver carrying the D766N mutation (R^766N^). (2) The VirA receiver (R_A_) formed a stable complex with the DNA-binding domain of VirG. R_A_ did not form a stable attachment to the DNA-binding domain of TorR, a response regulator which like VirG is a member of the OmpR/PhoB subclass of response regulators (Simon et al., 1994; Martinez-Hackert and Stock, 1997). (3) Glucose dose response assays in the presence or absence of the phenolic inducer acetosyringone (AS) indicated that VirAΔR and full-length VirA have similar dose responses to the ChvE-sugar signal, indicating that one function of the VirA receiver is to keep *vir* gene expression off when sugars are available, but no phenolic inducer is present.

## Materials and Methods

### Bacterial Strains and Plasmids

The bacterial strains and plasmids used in this study are listed in **Table 1.** Standard cloning methods were used for DNA ligation, PCR, plasmid isolation, and DNA analysis. Details of plasmid construction and primer sequences are included in **Supplementary Table S1**. *Escherichia coli* XL1-blue was used for plasmid construction and amplification. *E. coli* strain NEB Express I^q^ was used for protein expression for pull-down experiments. Use of a particular *Agrobacterium* strain for an experiment is indicated in the associated legend.

**Table 1:** Bacterial strains and plasmids used in this study.

Strains/plasmids	Relevant characteristics	Reference
***Escherichia coli***		
XL1-Blue	*recA1 endA1 gyrA96 thi-1 hsdR17 supE44 relA1 lac*[F’ *proAB lacIq ZM15 Tn10* (Tc^r^)]	Stratagene
NEB Express I^q^	Mini *F lacIq (cmR)/fhuA2 [lon] ompT gal sulA11 R(mcr-73::miniTn10)2 [dcm] endA/mcrC-mrr) 114::IS10*	New England Biolabs
***Agrobacterium tumefaciens***		
A348	C58 background with pTiA6NC	Lee et al., 1992
A348-3	A348 with *virA::kan*	Lee et al., 1992
A136	C58 cured of pTi	Watson et al., 1975
AB400	A348 with *virAΔR* (Δ707–829) replacing wt *virA* on pTi kan^R^	Wise et al., 2010
**Plasmids**		
pSW209Ω	Inc P plasmid that carries P*virB-lacZ* fusion	Lee et al., 1992
pAW10	Inc W *E. coli/Agrobacterium* shuttle vector has same parent plasmid as pYW15b.	Wise et al., 2005
pAW50	Low copy *E. coli/Agrobacterium* shuttle vector	Wise et al., 2005
pAW52	*virA* on 4.6 kb fragment at KpnI site of pAW50. pAW52 carries a FLAG tag at the C-terminus.	Wise et al., 2005
pYW15b	Inc W broad host range expression vector derived from pYW12 by including the constitutive P_N25_ promoter and the RGS-6X His tag.	Wang et al., 2000
pAW116	P_N25_-*virA*(Δ707-829). Includes FLAG tag at the C-terminus in PUC19.	This study
pAW117	KpnI fragment carrying PN25-*virA*ΔR-FLAG from pAW116 at the KpnI site of pAW10.	This study
pYW47	P_N25_*-virG* in pYW15b. Includes RGS-6X His tag at the N-terminus	Wang et al., 2000
pAW184	P_N25_*-virA* receiver domain (R_A_) in pYW15b. Includes receiver codons from V698 to T829 with RGS-6X His tag at N-terminus and FLAG tag at C-terminus.	This study
pAW187	P_N25_-DNAB *virG* DNA-binding domain (DNAB_V G_) in pYW15b. Includes RGS-6X His tag at the N-terminus.	This study
pAW192	P_N25_-*torR* DNA-binding domain (DNAB_TR_) from *E. coli torR* in pYW15b. Includes RGS-6X His tag at the N-terminus.	This study
pAW220	*virA*Δ^D766N^ with C-terminal FLAG tag in pUC19	This study
pAW221	*virA*^D766N^ derived from pAW220 in pAW50. pAW221 carries a FLAG tag at the C-terminus.	This study
pAW223	P_N25_-*virA*^D766N^ receiver domain (RD766N) in pYW15b. Includes receiver codons from V698 to T829 with RGS-6X His tag at N-terminus and FLAG tag at C-terminus.	This study
pRG109	Inc P plalsmid carrying P*virB-lacZ* and P_N25_-*virG.* Includes 6X His tag at the N-terminus.	Gao and Lynn, 2005
pAW97	*virA*Δ707-829 in pRG109, *virA*ΔR is expressed from the native promoter.	This study
pAW100	*virA* in pRG109, *virA* is expressed from the native promoter.	Wise et al., 2010


### β-Galactosidase Assays for Analysis of *vir* Gene Induction

*vir* gene induction was analyzed by triplicate measurements of β-galactosidase activity produced from a *virB-lacZ* fusion carried on the indicated plasmid. Overnight MG/L (Wise et al., 2006) cultures of *Agrobacterium* sp. were used to inoculate AB^∗^ (Wise et al., 2006) media containing 0.22% glycerol, 0.22% glucose, and/or AS as indicated. AB^∗^ culture samples were assayed according to the method of Miller (1972). Graphed values are the average of triplicate assays with the standard derivative indicated by error bars.

### VirA Receiver Domain Pull-Down Assays

The plasmid constructs used in the pull-down experiments are diagramed in **Figure 4.** NEB Express I^q^ cells carrying pAW184, pAW187, or pAW192 were grown in LB medium supplemented with 70 μg/ml ampicillin at 37°C to an OD600 = 0.5. Isopropyl-β-D-thio-galactopyranoside (Sigma) was added to 1 mM and incubation was continued at 25°C for 5 h. Cells were precipitated, washed and re-suspended in a lysis buffer consisting of 50 mM NaPO4 pH 7.6, 50 mM NaCl, 100 μg/ml lysozyme (Affymetrix), EDTA-free protease inhibitor (Roche), 20 μg/ml DNAse I, and 10 μg/ml RNAse A. Cells were opened with sonication, and lysates were cleared with centrifugation at 10 krpm for 10 min at 4°C. When desired, Ni-NTA agarose (Qiagen) was used to obtain purified proteins, as per Qiagen instructions. For pull-down experiments, purified protein and/or cleared lysate was mixed with anti-FLAG M2 affinity gel (Sigma) in a TBS buffer consisting of 50 mM Tris-HCl pH 7.5, 75 mM NaCl, 1 mM EDTA, and 0.8% TritonX-100. Incubation with mixing was done in 2 ml micro-centrifuge tubes overnight at 4°C. Following incubation, samples were centrifuged to precipitate the FLAG affinity gel. A supernatant sample was reserved from this initial precipitation. The affinity gel with bound proteins was then washed three times with TBS. To avoid collecting unbound proteins that precipitated along with the affinity gel, the gel was transferred to Pierce Spin cups with paper filter (Thermo-Scientific) for three additional washes. Protein was eluted from the affinity gel according to the method recommended by Sigma using SDS-PAGE sample buffer without a reducing agent (60 mM Tris-HCl pH 6.8, 2% SDS, 10% glycerol, 0.002% bromophenol blue).

### Immunoblot Analysis

β-mercaptoethanol was added to pull-down eluates to 5%. Samples were heated 20 min at 85°C and equivalent volumes were loaded on a 15% SDS-polyacrylamide gel. Proteins were visualized by Western blot analysis with anti-RGS-His antibody (Qiagen), ECL anti-mouse horseradish peroxidase-linked antibody (GE Healthcare) and ECL Western Blotting Detection reagent (Amersham).

## Results

### Constitutive Expression of *virA*ΔR Corrects the Null Phenotype of *virA*ΔR Expressed from the Ti Plasmid

**FIGURE 2:**
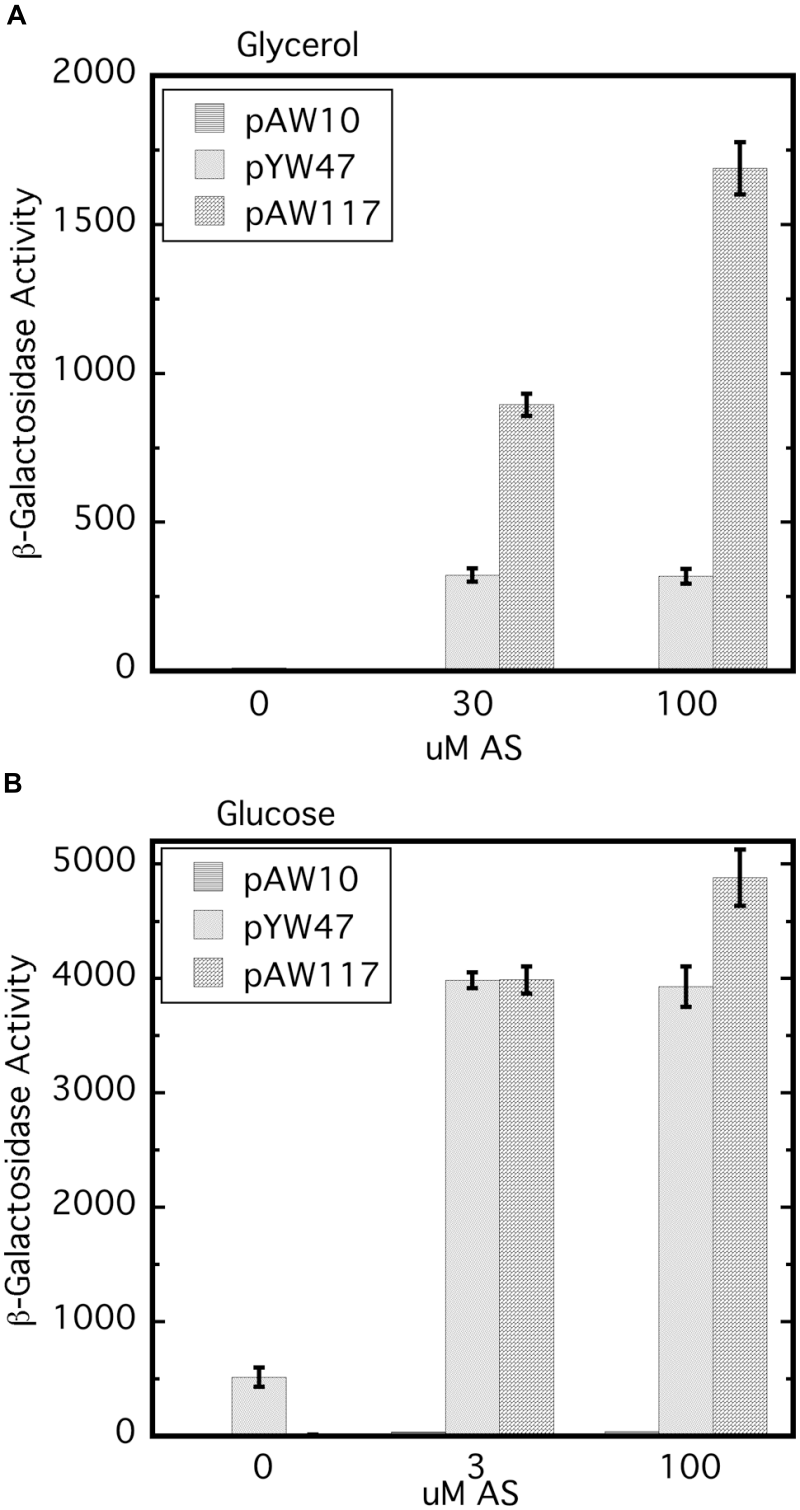
**The null phenotype of AB400 (*virA*ΔR on pTi) can be overcome by constitutive expression of either *virA*ΔR or *virG*.** AB400/pSW209Ω (*virB-lacZ*) carrying pAW10 (vector), pYW47 (P_N25_-*virG*) or pAW117 (P_N25_-*virA*ΔR) were assayed for *vir* gene expression. **(A)** AB^∗^ induction medium included glycerol as the carbon source and 0, 30, or 100 μM AS. Student’s *t*-test indicates that pAW47 and pAW117 containing strains are different from one another (*p* > 0.95). **(B)** Induction medium included glucose in addition to glycerol and 0, 3, or 100 μM AS.

Replacement of wild type *virA* on pTi with *virA*ΔR (AB400) creates a null phenotype, even though the strain carries wild type *virG* at the native location (Wise et al., 2010). In this case because *virA* and *virG* are under the control of their own auto-inducible promoters, the levels of both VirA and VirG are expected to be extremely low (e.g., see Lee et al., 1992; Wise et al., 2010). As shown previously, constitutive expression of *virG* in AB400 (Wise et al., 2010) or A136 (Chang and Winans, 1992) allows VirAΔR to function. **Figure 2** shows that the null phenotype of AB400 can also be corrected by constitutive expression of *virA*ΔR (pAW117). In fact, in the glycerol medium, *vir* gene expression was significantly higher with constitutively expressed *virA*ΔR than with constitutively expressed *virG* (pYW47). In medium containing glucose and AS, *vir* gene expression was similar whether the cells constitutively expressed *virG* or *virA*ΔR, with the aforementioned exception of constitutively expressed *virG* bypassing the requirement for AS. The phenotype resulting from constitutive expression of *virA*ΔR contrasted with the phenotype previously observed when *virA*ΔR is expressed from its native promoter on the same multi-copy vector in the *virA* deletion strain, A348-3 (Wise et al., 2010). In that case, *virA*ΔR was inactive unless the media included both sugar and a phenolic inducer. The variable results indicate that the initial concentrations of VirA and VirG are prime determinants of the ultimate *vir* gene induction phenotype. A summary table of various strains, their genotypes and *vir* gene expression phenotypes is presented in **Supplementary Table S2**.

### Expression of the VirA Receiver *In Trans* does not Correct the Null Phenotype of AB400

**FIGURE 3:**
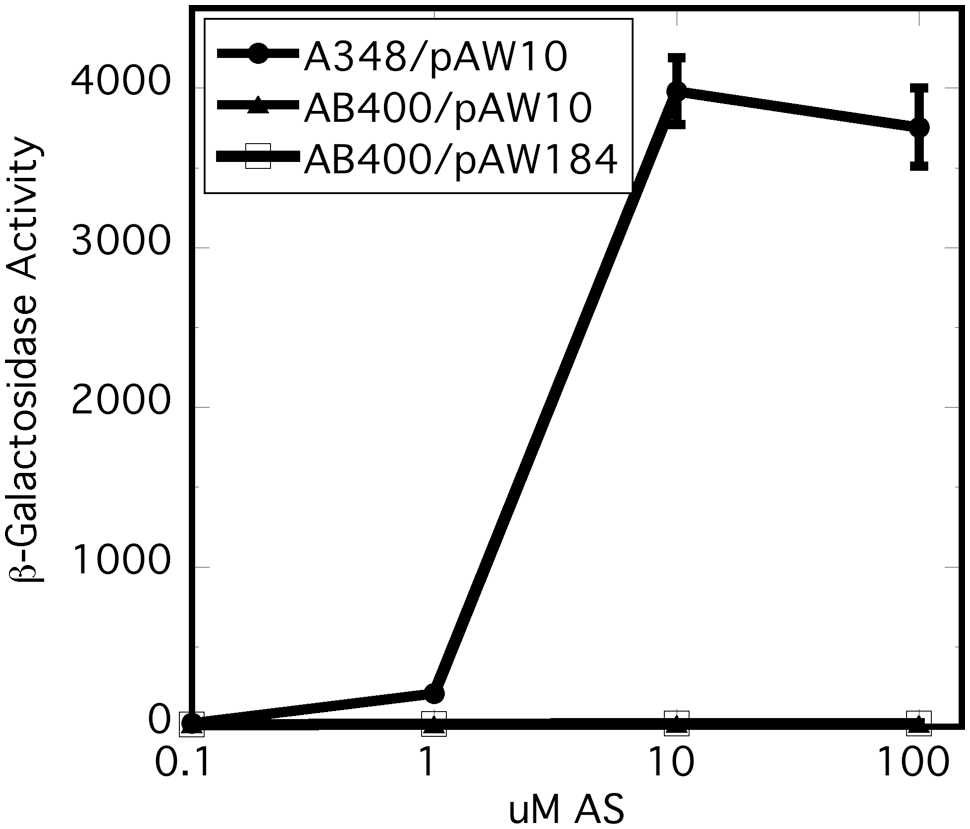
**Expression of the *virA* receiver domain *in trans* did not correct the null phenotype of AB400.** A348 carries wild type *virG* and wild type *virA* on pTi. AB400 carries wild type *virG* and *virA*ΔR on pTi. pAW10 is an empty vector. pAW184 carries constitutively expressed *virA* receiver domain (see **Figure 4**). Cells were grown in AB^∗^ induction medium containing glucose and the indicated amounts of acetosyringone. Both AB400 and A348 carried the pSW209Ω (*virB-lacZ*) plasmid.

*In vitro* experiments have shown that the receiver domain of the *Rhizobium leguminosarum* hybrid kinase FixL is phosphorylated when expressed *in trans* to a version of FixL that carries an intact kinase, but no receiver (Boesten and Priefer, 2004). We reasoned that, if the receiver domain of VirA plays a role in phosphate relay to VirG, expression of the domain *in trans* could conceivably correct the null phenotype of AB400. However, constitutive expression of the receiver (pAW184) had no effect on *vir* gene expression in the AB400 background (**Figure 3**). The receiver domain expressed from pAW184 was shown to have biological activity through its ability to inhibit *vir* gene expression when included in the wild type background of A348 (see below). We concluded that the receiver must be attached to the remainder of the VirA protein in order to function as a positive factor for *vir* gene expression. This result does not disprove a role in phosphorylation for the receiver, but is consistent with a VirG recruiting function.

### The VirA Receiver Domain Forms a Stable Complex with the VirG DNA-Binding Domain

*virA*ΔR expressed from pTi created a situation where the proteins could not functionally interact unless the cellular content of one of the proteins was artificially increased (**Figure 2**). Several lines of evidence suggest that the receiver domain is not involved in a phospho-relay (Jin et al., 1990; Pazour et al., 1991; Mukhopadhyay et al., 2004; Wise et al., 2010). In addition, analysis indicates that hybrid histidine kinases have evolved independently through lateral recruitment (Zhang and Shi, 2005), suggesting possible functional variations. These considerations led to the hypothesis that the VirA receiver could act as a recruiting factor for VirG.

**FIGURE 4:**
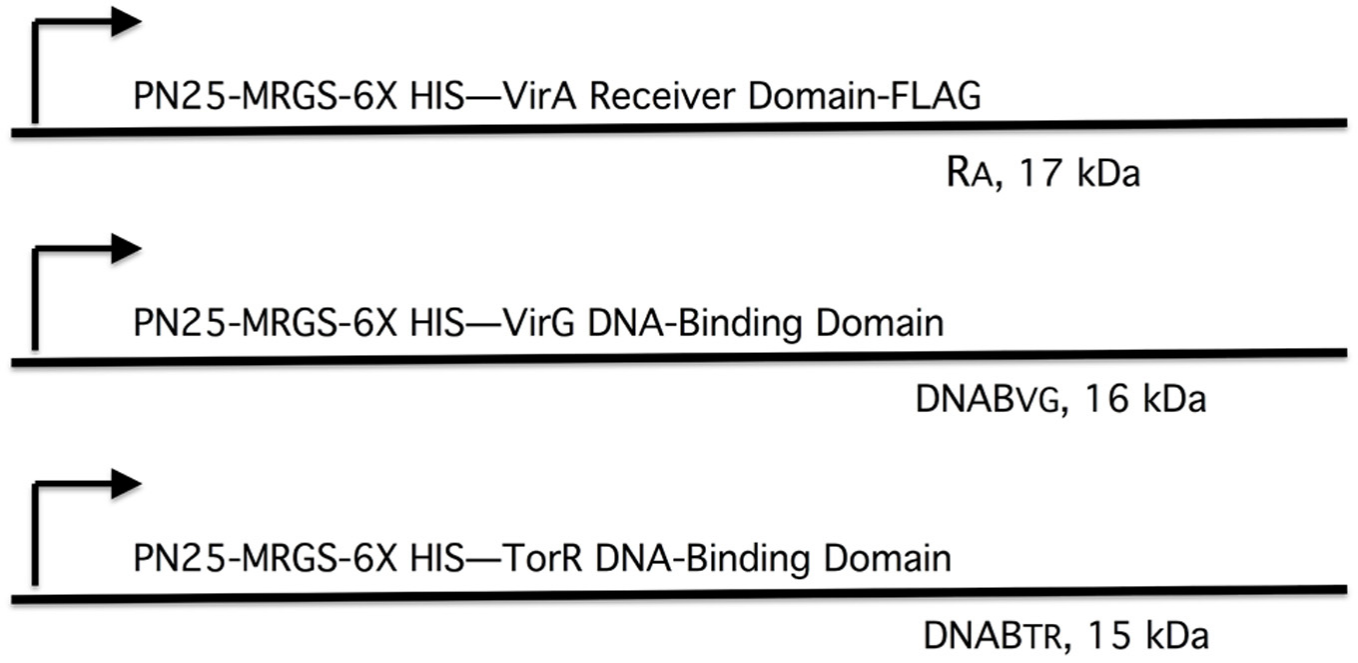
**Plasmid constructs used in pull-down assays.** The plasmids are derivatives of pYW15b (Wang et al., 2000). The VirA receiver domain (R_A_) is constitutively expressed from P_N25_ and includes a FLAG tag at the carboxyl end (pAW184). The VirG DNA-binding domain (DNAB_V G_) and the TorR DNA-binding domain (DNAB_TR_) are constitutively expressed from P_N25_ (pAW187 and pAW192, respectively). Each construct carries an RGS-6XHis tag near the amino terminus. The molecular weight, including epitope tags, is indicated for each protein fragment.

We tested this hypothesis with pull-down assays using the constructs shown in **Figure 4.** The three constructs, *virA* receiver domain (R_A_, pAW184), *virG* DNA-binding domain (DNAB_V G_, pAW187), and the *E. coli torR* DNA-binding domain (DNAB_TR_, pAW192) are all derivatives of pYW15b (Wang et al., 2000). TorR, like VirG, is included in the OmpR class of response regulators (Martinez-Hackert and Stock, 1997) and its DNA-binding domain was included as a control for non-specific binding. Each plasmid construct constitutively expresses the protein fragment with an RGS-6XHis tag at the N-terminus. In addition, the *virA* receiver construct is FLAG-tagged at its C-terminus. Cell lysates and, where indicated, Ni-NTA purified proteins were used in pull-down reactions that contained FLAG-affinity gel. (see Materials and Methods).

**FIGURE 5:**
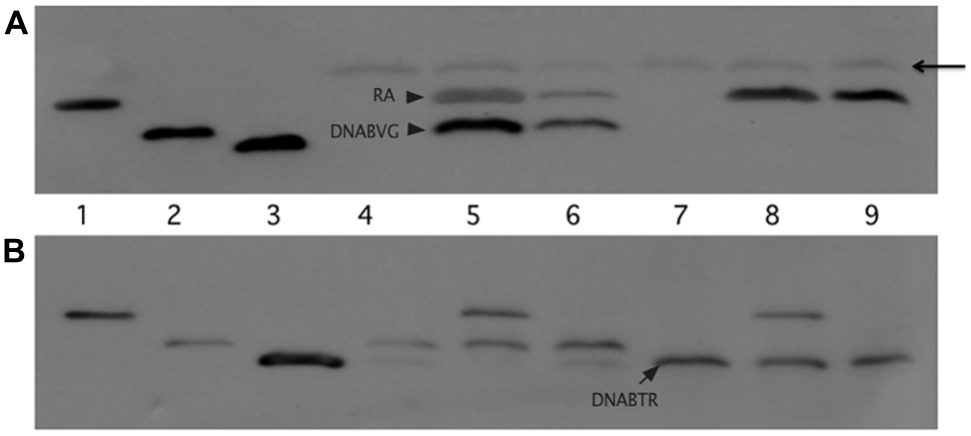
**The VirA receiver domain binds to the VirG DNA-binding domain, but not to the DNA-binding domain of TorR.** The first three lanes of both immunoblots show Ni-NTA purified proteins: lane 1, VirA receiver domain; lane 2 VirG DNA-binding domain; lane 3 TorR DNA-binding domain. **(A)** Top blot, lanes 4 through 9 were loaded with the eluates of the pull-down assays. Each reaction included equivalent amounts of FLAG-affinity gel and lysate from cells that produced the VirG DNA-binding domain from pAW187 (lanes 4, 5, and 6) or lysate from cells that produced the TorR DNA-binding domain from pAW192 (lanes 7, 8 and 9). Lanes 5 and 8 included lysate from cells that produced the VirA receiver domain (pAW184). Lanes 6 and 9 included lesser amounts of purified VirA receiver domain. The arrowhead indicates light chain antibody derived from the anti-FLAG affinity gel. **(B)** The lanes in the lower blot correspond to the lanes in blot **(A),** but lanes 4 through 9 were loaded with samples of supernatant recovered after the first precipitation of the affinity gel from the pull-down reactions, before washing. The supernatant samples show that significant amounts of DNAB_TR_ had been added to the pull-down reactions (**A**, lanes 7, 8 and 9) and remained in the supernatant (**B**, lanes 7, 8, and9).

**Figure 5** depicts the results of the pull-down experiments. In **Figures 5A,B**, the first three lanes (R_A_, DNAB_V G_, and DNAB_TR_) are purified proteins acting as size markers. The reaction loaded in lane 4 (**Figure 5A**) contained only lysate from cells that expressed the VirG DNA-binding domain (DNAB_V G_). As expected, the FLAG affinity gel was unable to pull-down any protein from this reaction. Additional reactions, included in addition to lysate from cells expressing DNAB_V G_, either lysate from cells expressing the VirA receiver domain (R_A_) or purified receiver domain (lanes 5 and 6, respectively). In both cases, the VirG DNA-binding domain was pulled down when the reaction mix included the VirA receiver domain. This was true despite extensive washing to remove proteins not bound to the affinity gel, indicating that the interaction between the VirA receiver and the VirG DNA-binding domain was stable under these conditions. A reaction that included lysate from cells expressing the TorR DNA-binding domain (DNAB_TR_) along with the affinity gel (lane 7) pulled down no protein. The addition of VirA receiver domain lysate (lane 8) or purified receiver domain (lane 9) did not pull-down the TorR DNA-binding domain. **Figure 5B** shows supernatant from the initial precipitation (see Materials and Methods) of FLAG affinity gel following incubation. Supernatant loaded in lanes 7 and 9 indicate that purified R_A_ was more limited in those reactions than R_A_ present in whole lysates (lanes 5 and 8) and, thus only appears in the pull-down reaction, not the more dilute supernatants. Lanes 8, and 9 (**Figure 5B**) indicate that while DNAB_TR_ was included in the reactions containing R_A_, it remained in the supernatant. The fact that the VirA receiver did not bind to the DNA-binding domain of TorR indicates that the receiver has some specificity for the DNA-binding domain of its cognate response regulator VirG.

### Inhibition of *vir* Gene Expression by the Wild Type VirA Receiver is Stronger than Inhibition by the D766N Mutant Receiver in the A348 Wild Type Background

**FIGURE 6:**
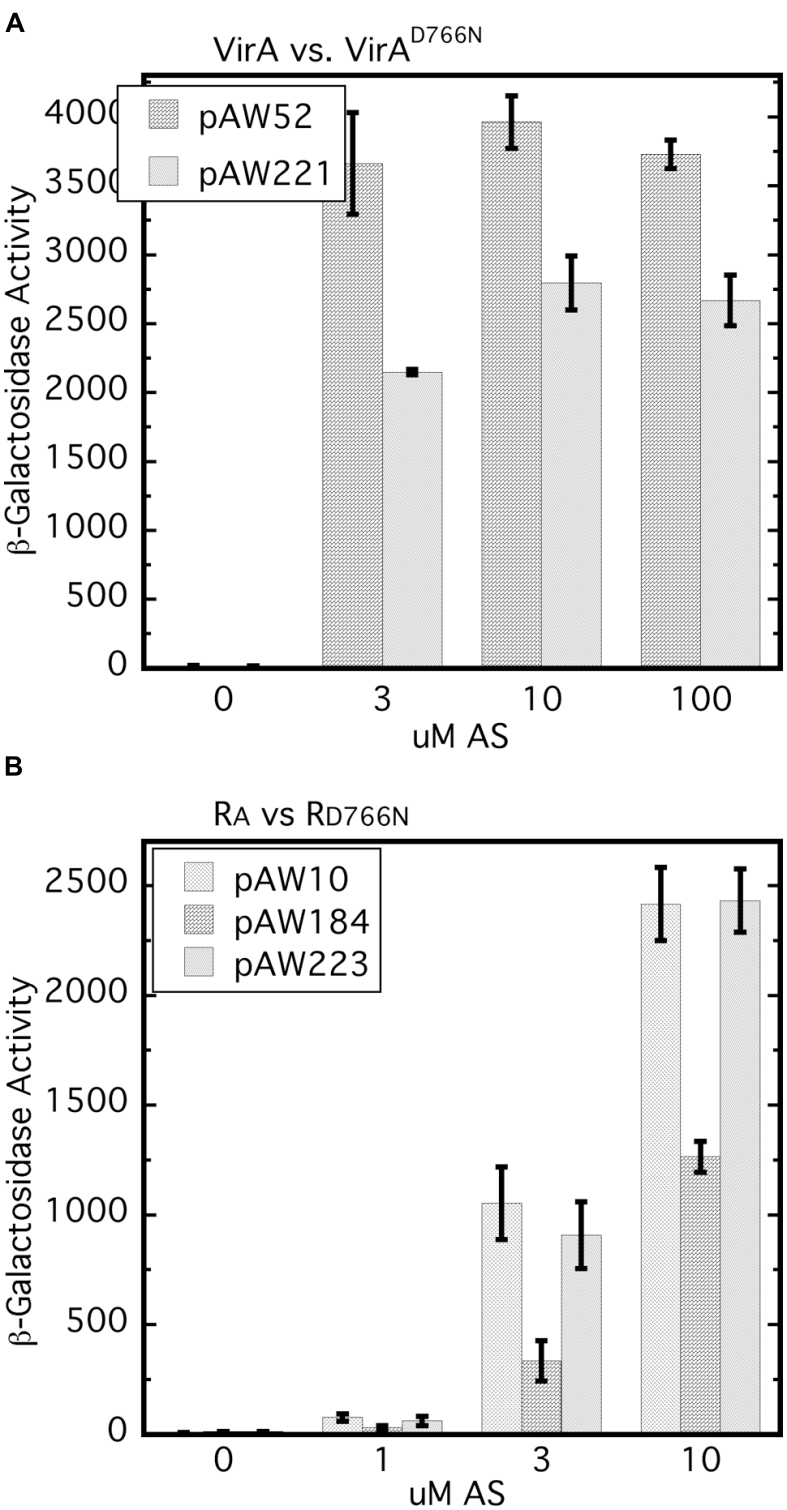
**The D766N receiver mutation reduces *vir* gene expression and is less inhibitory than the wild type receiver.** **(A)** *vir* gene expression that relies on VirA_D766N_ is compared with that which depends on wild type VirA. A348-3 (Δ*virA*) carrying pSW290Ω (*virB-lacZ*) and pAW52 (*virA*) or pAW221 (*virA*_D766N_) were grown in AB^∗^ induction medium with 0.22% glucose and the indicated amounts of AS. Student’s *t*-test showed that at 3, 10, and 30 μM AS VirAD766N is different than VirA (*p* > 0.95). **(B)** A348/pSW209Ω carrying pAW10 (vector), pAW184 (P_N25_-R_A_), or pAW223 (P_N25_-R_D766N_) was grown in AB^∗^ medium containing 0.22% glucose and AS. The Tukey test for these three strains indicated that A348/pSW209Ω/pAW184 is different than same strain with pAW10 or pAW223 (*p* > 0.95) and that pAW223 and pAW10 are not different (*p* > 0.95).

It has been reported previously that the D766N receiver mutation reduces *vir* gene expression by approximately 80% (Pazour et al., 1991). In our hands, VirA_D766N_ expressed from a low copy vector (pAW221) in A348-3 (Δ*virA*) reduced *vir* gene expression by 20–40% relative to wild type VirA (**Figure 6A**). The different results may be due to differences in plasmid copy number and strain variation. A348-3 carries wild type *virG* in the native position on the Ti plasmid whereas in the earlier work both *virG* and *virA* were expressed from a multi-copy plasmid in A136.

An earlier study showed that constitutive expression of the *virA* receiver domain in *trans* resulted in restoration of repression of *vir* gene expression in a strain carrying *virA*ΔR and constitutively expressed *virG* (Chang et al., 1996). Moreover, this repressive *trans* activity of the receiver domain was observed when it carried the D766N mutation. Because our work has shown the importance of the initial concentration of VirG we compared *vir* gene expression in the wild type background (A348) for cells that constitutively expressed either the wild type receiver (pAW184) or R_D766N_ (pAW223), or carried the empty vector pAW10. The wild type receiver consistently repressed *vir* gene expression in the wild type background (**Figure 6B**). In several repetitions of this assay, R_D766N_ had modest but not significant repressive activity while being expressed at a similar level as the RA construct (**Supplementary Figure S1**). Thus the inhibitory effect, which presumably depends on the ability of R_A_ to sequester VirG through interaction with its DNA-binding domain, appears to be dependent on D766N. Interestingly, and consistent with Chang et al. (1996), R_D766N_ seemed almost as inhibitory as R_A_ when A348 also carried P_N25_-*virG* (**Supplementary Figure S2**). Taken together these results further emphasize that the initial VirG concentration affects its interactions with the VirA receiver domain and the effects of that domain on *vir* gene expression.

### Glucose Dose Response Assays Show that the VirA Receiver Domain Regulates Phosphate Transfer to VirG and Suggests that the ChvE-Dependent Sugar Signal Increases Auto-Phosphorylation

**FIGURE 7:**
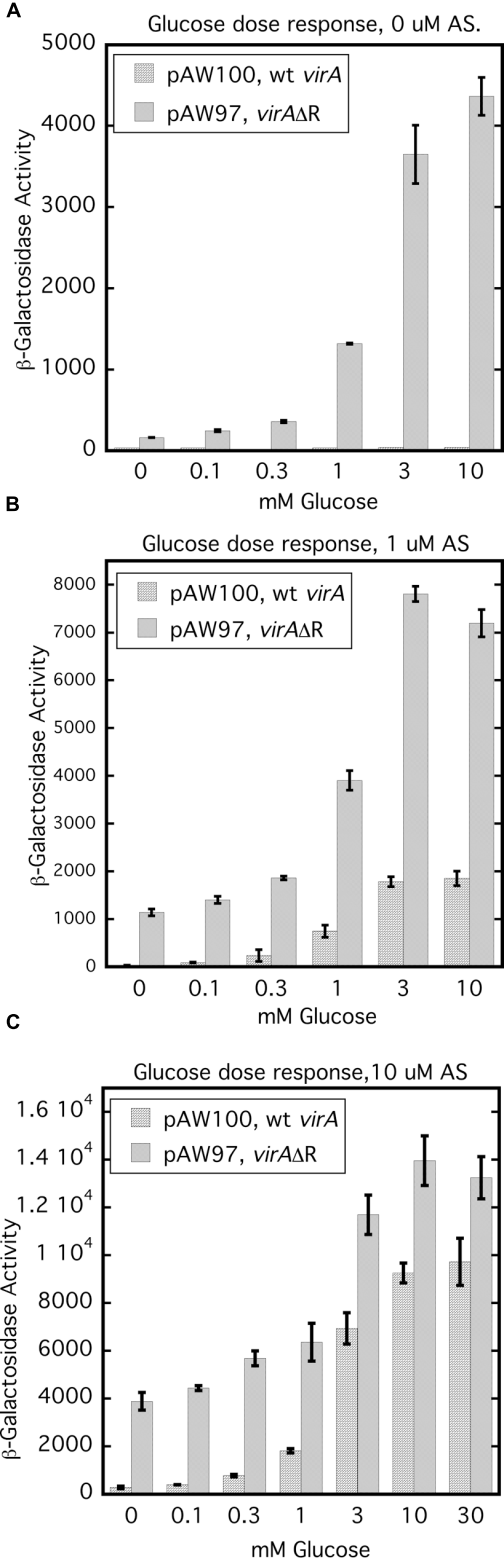
**Glucose dose response assays indicate that the ChvE-dependent sugar signal affects a function of VirA that is partly independent from the effect of the phenolic inducer.** A136 (no pTi) carrying either pAW100 (wt *virA*, P_N25_-*virG*, *virB-lacZ*) or pAW97 (*virA*ΔR, P_N25_-*virG*, *virB-lacZ*) were assayed in AB^∗^ induction medium with increasing concentrations of glucose. **(A)** Induction media did not contain AS. **(B)** Induction media contained 1 μM AS. **(C)** Induction media contained 10 μM AS.

Removal of the receiver domain of VirA increases activity of that protein, provided the cellular content of VirG is increased (Chang and Winans, 1992; Chang et al., 1996; Brencic et al., 2004; Wise et al., 2010). We investigated the repressive nature of the receiver domain by comparing the activity of full-length wild type VirA with that of VirAΔR in the presence of constitutively expressed *virG* in a glucose dose response assay. **Figure 7** represents the results of glucose dose response assays done with 0, 1, and 10 μM AS. For this experiment, A136 carried either pAW97 (P_N25_-*virG*, *virA*ΔR, *virB-lacZ*) or pAW100 (P_N25_-*virG*, *virA*, *virB-lacZ*). In **Figure 7A**, cells were grown with increasing amounts of glucose, but no phenolic inducer. Predictably, full-length VirA was inactive, while VirAΔR-dependent *vir* gene expression increased with the concentration of glucose. When 1 μM AS was included in the growth medium, VirAΔR had some activity in the absence of sugar (0 mM glucose) and full-length VirA became functional as the concentration of glucose was increased (**Figure 7B**). Increasing the AS concentration to 10 μM further increased *vir* gene expression for both strains (**Figure 7C**). Although VirAΔR could respond to either the phenolic inducer or glucose, full-length VirA required sugar for activity at these relatively low concentrations of AS (1 or 10 μM). However, half maximal expression occurred between 1 and 3 mM glucose for VirAΔR, regardless of the presence of AS. Half maximal activity was also between 1 and 3 mM glucose for full-length VirA if AS was present in the assay medium. Thus, the half-maximal response to glucose appeared to be independent of AS concentration and the presence of the receiver, while the primary effect of increased AS concentration for both strains appeared to be an increase in maximum *vir* gene expression at a particular glucose concentration. These observations suggest that the ChvE-dependent sugar signal and the phenolic inducer affect two functions of the protein that are largely separate.

## Discussion

*Agrobacterium* tightly controls expression of the virulence genes to conserve energy and resources until an appropriate host plant is detected. VirA and VirG are both essential for virulence, but their interdependent and self-regulated expression (Winans et al., 1988) means that these proteins are present in miniscule amounts in the early stages of induction (Lee et al., 1992; Wise et al., 2010). Removing the receiver domain from VirA, as in AB400, appears to create a situation where productive encounters between VirA and VirG are too infrequent to up-regulate the *vir* gene control system. The null phenotype of AB400 could be overcome by increasing the cellular content of either VirAΔR or VirG (**Figure 2**). This finding likely reflects a situation whereby increasing the concentration of either of the proteins permits them to interact with sufficient frequency to further induce expression of the *virA*ΔR and *virG* genes located on the Ti plasmid. Early experiments identified the receiver domain of VirA as an inhibitory factor: the normally essential phenolic inducer was unnecessary for gene expression mediated by VirAΔR. Those experiments were done with cells that constitutively expressed *virG* (Chang and Winans, 1992). In contrast, *vir* gene expression that depended on P_N25_-*virAΔR* in the AB400 background (**Figure 2**) did require the phenolic inducer even with glucose in the medium. This difference may reflect the fact that overexpressed VirAΔR is membrane-bound with limited mobility compared to the cytoplasmic VirG protein.

Our pull-down assays indicated that the receiver domain of VirA forms a stable complex with the DNA-binding domain of VirG. This complex formation is the probable determinant of both the receiver’s positive effect on *vir* gene expression and the explanation for the inhibitory effect found when the receiver is expressed in trans. Mechanisms that contribute specifically to the interaction of cognate regulatory proteins have been more studied in eukaryotes (Pawson and Nash, 2003), but a few have been identified in prokaryotes. Prokaryotic kinases that carry domains that function specifically as recruiting or docking factors for their regulatory partners include RcsD (Schmöe et al., 2011), BglF (Lopian et al., 2003), CikA (Zhang et al., 2006), and CheA (McEnvoy et al., 1996).

The interaction between the VirA receiver domain and the VirG DNA-binding domain has similarities in the Rcs regulatory system. The RcsD protein transfers a phosphate from an Hpt domain to RcsB, the system’s response regulator. Efficient phosphate transfer is contingent on stabilization of the RcsD/RcsB interaction and that stabilization is achieved through the interaction of an RcsB structural domain (ABL) with the DNA-binding domain of the response regulator (Schmöe et al., 2011). Perhaps, the role of response regulator recruitment should be considered for other hybrid kinases.

Brencic et al. (2004) examined inhibition of *vir* gene expression in merodiploids that expressed full-length or VirAΔR alleles in strains that also carried versions of VirA that were constitutively active in the absence of a phenolic inducer. The authors concluded that inhibitory activity depended on the receiver domain. Here, we have looked at *vir* gene inhibition in the wild type background of A348. Expression of R_A_ *in trans* had a distinct inhibitory effect in the wild type background of A348. We suspect that the inhibition seen by Brencic et al. (2004) and in **Figure 6B** is due to detached receiver protein interacting with the VirG DNA-binding domain, thereby reducing the availability of VirG for phosphorylation by an intact VirA protein. We hypothesize that transfer of a phosphate group from the VirA kinase to the VirG receiver domain would release full-length VirA from VirG and allow activated VirG to bind the *vir* gene promoters.

Interestingly, R_D766N_ was less inhibitory than the wild type receiver in the wild type background of A348, but nearly equivalent when *virG* was expressed from the constitutive PN25 promoter, the latter result being consistent with those of Chang et al. (1996). In our assays the D766N mutation in full-length VirA expressed from a low copy plasmid has a consistent negative effect on *vir* gene expression in induction medium containing glucose. It may be that both the reduced *vir* gene expression mediated by VirA_D766N_ and the reduced inhibition by R_D766N_ in A348 stem from a slightly reduced affinity of those proteins for the VirG DNA-binding domain. However, we were unable to show a clear difference in the affinity of R_A_ and R_D766N_ for the DNA-binding domain of VirG using pull-down assays. The basis for the observation that the inhibitory capacity of R_D766N_ was nearly equivalent to R_A_ in the presence of constitutively expressed *virG* is, at this point, not clear. Perhaps the high levels of VirG may drive the formation of VirG/R_D766N_ complexes thereby reducing the level of VirG available for phosphorylation by VirA, but further exploration of various models is needed.

The glucose dose response experiments (**Figure 7**) demonstrated that the concentration of AS had no effect on the half-maximum values for *vir* gene expression which was consistently between 1 and 3 mM glucose. These results imply that the ChvE-sugar signal influences a function of the VirA protein that is independent from the effect of the phenolic inducer. VirAΔR’s ability to activate VirG when phenolics are absent may reflect increased auto-phosphorylation stimulated by the sugar signal. Provided the cellular concentration of VirG is sufficiently elevated, the absence of the VirA receiver allows the phosphate group to be easily transferred to VirG. A ChvE-sugar dependent increase in auto-phosphorylation is also consistent with the strong increase in *vir* gene expression driven by wild type VirA in medium that contains both a phenolic inducer and an enhancing monosaccharide relative to the significantly lower activity observed if glycerol replaces the sugar as carbon source. In the case of full-length VirA, a phenolic inducer is required, apparently, to counter the obstruction of phosphate transfer that defines an inhibitory function of the receiver. An alternative model is that the phenolic signaling alters the ‘residence’ time of VirG on VirA, thereby affecting activation of VirG.

As a soil bacterium, *Agrobacterium* competes with numerous other microbes for nutrients. Soil can be a rich source of carbohydrates depending on regional micro-biota and the availability of root exudates (Kögel-Knabner, 2002). *Agrobacterium* carries redundant sugar transport systems that help in the competition for resources (Wood et al., 2001; Zhao and Binns, 2014). A variety of phenolics, derived from decaying lignin, are potential inducers of virulence and may also exist in soil. However, phenolics in the environment tend to be less stable than plant derived sugars (Spielvogel et al., 2007). As more than 20 genes are involved in the virulence process, it is in the best interest of the bacterium to strictly limit their induction to the plant environment where a productive infection may result in a nutritional source. Thus, the VirA receiver domain appears to have a role, not only in recruiting VirG, but also in limiting VirG activation to conditions that mimic those found at the site of a plant wound.

## Conflict of Interest Statement

The authors declare that the research was conducted in the absence of any commercial or financial relationships that could be construed as a potential conflict of interest.
