# Supplementary material for: The Receiver of the Agrobacterium tumefaciens VirA Histidine Kinase Forms a Stable Interaction with VirG to Activate Virulence Gene Expression
Source: Front Microbiol. 2016 Jan 8;6:1546. doi: 10.3389/fmicb.2015.01546 (PMC4705274; doi:10.3389/fmicb.2015.01546)
Supplement: Supplementary file 1 [file Presentation_1.PDF]

Supplementary Material

The receiver of the Agrobacterium tumefaciens VirA histidine kinase forms a stable interaction with VirG to activate virulence gene expression

Arlene A. Wise and Andrew N. Binns*

***Correspondance:** Corresponding Author: abinns@sas.upenn.edu

I. Supplementary Methods

Plasmid construction.

pAW97 carries *virA*ΔR (Δ707-829) from pAW82 (Wise et al., 2010) at the KpnI site of pRG109 (P_{N25}-*virG*, P*virB-lacZ,*Gao and Lynn, 2005).

pAW116(P_{N25}-*virA*Δ707-829-FLAG in pUC19) was created from pAW82 (Wise et et al., 2010) using sequential PCR to add a FLAG tag at the C-terminus. Primer ΔRFLAGm was used with primer 4120c and primer ΔRFLAGc was used with primer 3310m in two initial PCR reactions. The resulting PCR products were combined in a third self-annealing PCR reaction with primers 3310m and 4120c. This final PCR product was digested with SnaBI and EcoRV and used to place the FLAG tag and 2 stop codons at the end of the *virA* receiver domain in pFF5 (P_{N25}-*virA*, Wise et al., 2010). The KpnI fragment carrying P_{N25}-*virA*ΔR-FLAG was then cloned into pUC19 to give pAW116.

pAW117(PN25-*virA*ΔR, FLAG-tagged) was created by cloning theKpnI fragment from pAW116 into pAW10. The C-terminal FLAG epitope tag does not have a significant effect onthe activity of full-length VirA or VirAΔR.

pAW184 (P_{N25}-R_A-FLAG) carries the *virA* receiver domain sequences from codon V698 t o T829 with a FLAG epitope tag immediately upstream from the first of two stop codons. ASacI-SalI fragment was derived using PCR and primers 3460m and 3940c with pAW116 as template. The PCR fragment was initially cloned into pGEM-T (Promega) vector, then removed with a SacI-SalI digest and cloned into pYW15b (Wang et ., 2000). pYW15b addedP_{N25} and an MRG-6XHis tag to the N-terminus of the protein fragment.

pAW187 (P_{N25}-$DNAB_{VG}$) carries the *virG* DNA-binding domain sequences from codon V119 to A241. pAW187 was constructed using PCR with primers DNABVGm and DNAVc and template pYW47. The PCR fragment was initially cloned into pGEM-T, removed with a SacI-SalI digest and cloned into pYW15b, adding P_{N25} and an MRG-6XHis tag to the N-terminus.

pAW192 (P_{N25}-$DNAB_{TR}$) carries the *torR* DNA-binding domain sequences from L116 to C230. pAW192 was constructed using PCR and primers DNABTRm and DNABTRc with plasmid pMR32 (Roggiani and Goulian, 2015). The PCR f

ragment was cloned into pGEM-T, removed with a SacI digest and cloned into pYW15b to add P_{N25}-MRG-6XHis to the N-terminus.

pAW220 (*virA*$_{D766N}$-FLAG in pUC19) was constructed using sequential PCR and pAW19 (Wise et al., 2010) as template. Primer D766Nm was used with primer pUC500c and primer D766Nc was used with primer 3310m for two initial PCR reactions. The initial PCR products were used in a third self-annealing PCR r eaction with primers 3320m and pUC500c. This final PCR product was digested with DraIII and BspEI and used to replace the DraIII-BspEI fragment of pAW19.

pAW221(*virA*Δ^{766N}-FLAG) was constructed by cloning the pAW220 KpnI fragment carrying *virA*^{D766N} into pAW50.

pAW223(P_{N25}-R^{D766N}-FLAG) was constructed using the same method and primers used for pAW184, but with pAW220 as template.

Restriction and sequencing analysis confirmed the accuracy of all plasmid constructs.

II. Supplementary Tables

Table S1. Primers used in plasmid construction.

primer	sequence	notes
3310m	5'-ACCCCACATTTTTGAACCCTTC-3'	match to sequence upstream of *virA* receiver
4120c	5'-ACAACAGCCCGTCGTCCACAC-3'	complement downstream of *virA* receiver
3460m	5'-CTTCTAAGGAGCTCGTAAATCCAGACAG-3'	adds a SacI site upstream of *virA* receiver
3940c	5'-CTTGATCCACTGAACCTATGCCGTTCGATC-3'	complement downstream of *virA* gene
DNABVGm	5'-CCCAACGAGCTCCGCTCCAAAGACC-3'	adds a SacI site upstream of *virG* DNA-binding domain
DNABVGc	5'-CCATCGTCCCCCGGGCGAAACCTGC-3'	complement downstream of *virG* gene
DNABTRm	5'-GTGAAAAATGAGCTGTGGCGAATCGAC-3'	adds a SacI site upstream of *torR* DNA-binding domain
DNABTRc	5'-AGCTTGCATGCCTGCAGGTCGAC-3'	complement downstream of *torR* gene
D766Nm	5'-CTGGTCATGGTCAACCAAGCGTCTCTTC-3'	addsD766N mutation
D766Nc	5'-GAAGAGACGCTTGGTTGACCATGACCAG-3'	addsD766N mutation
pUC500c	5'-GTTGTGTGGAATTGTGAGCG -3'	complement pUC19 downstream of *virA* insert
ΔRFLAGm	5'-GACTACAAGGACGACGATGACAAGTAGTAGGCACCG CGTGGAAACGG-3'	match to pAW82, adds FLAG before stop codons
ΔRFLAGc	5'-CTTGTCATCGTCGTCCTTGTAGTCGCGGCCGAAAAAACTGTCTGGATTTAC-3'	complement to pAW82, adds FLAG before stop codons

Table S2.*vir* gene expression variation and strain/plasmid characteristics.

**Strain (plasmid)**	**plasmid description**	**Strain's pTi characteristics**	**vir gene expression**	**Figure/ref.**
**AB400**		***virA*ΔR and wt*virG* on pTi**		
(pAW10)	vector		none<br>null phenotype	Fig. 2<br>This study
(pAW117)	P_{N25}-*virA*ΔR		requires AS	Fig. 2<br>This study
(pYW47)	P_{N25}-*virG*		requires sugar or AS	Fig. 2<br>This study
**A136**		**no pTi**		
(pCH116d1)	Plac-*virG*, *virA*ΔR		requires sugar or AS	Chang and Winans 1992
(pAW116d2)	Plac-*virG*, *virA*		requires AS	Chang and Winans, 1992
(pAW97)	P_{N25}-*virG*, *virA*ΔR		requires sugar or AS	Fig. 7<br>This study
(pAW100)	P_{N25}-*virG*, *virA*		requires AS	Fig. 7<br>This study
**A348-3**		**Δ*virA*::kan, wt*virG* on pTi**		
(pAW16)	*virA*		requires AS	Wise et al., 2010
(pAW102	*virA*ΔR		requires sugar and AS	Wise et al., 2010
(pAW103	P_{N25}-*virG*, *virA*ΔR		requires AS or sugar	Wise et al., 2010
(pYW47)	P_{N25}-*virG*		none, null phenotype	Wise et al., 2010

Key observations for Table S2: Due to the auto-regulatory nature of *virA* and *virG*, *vir* gene expression is sensitive to the cellular content of VirA and VirG at the early stages of induction. VirAΔR is unable to activate VirG unless the level of VirAΔR or VirG is increased either through constitutive expression (Plac, P_{N25}) or inclusion of the gene(s) on a multi-copy plasmid. Note: overexpression of *virG* (P_{N2}5-*virG*) in the absence of VirA does not induce *vir* gene expression, regardless of the presence of AS.

III. Supplementary Figures

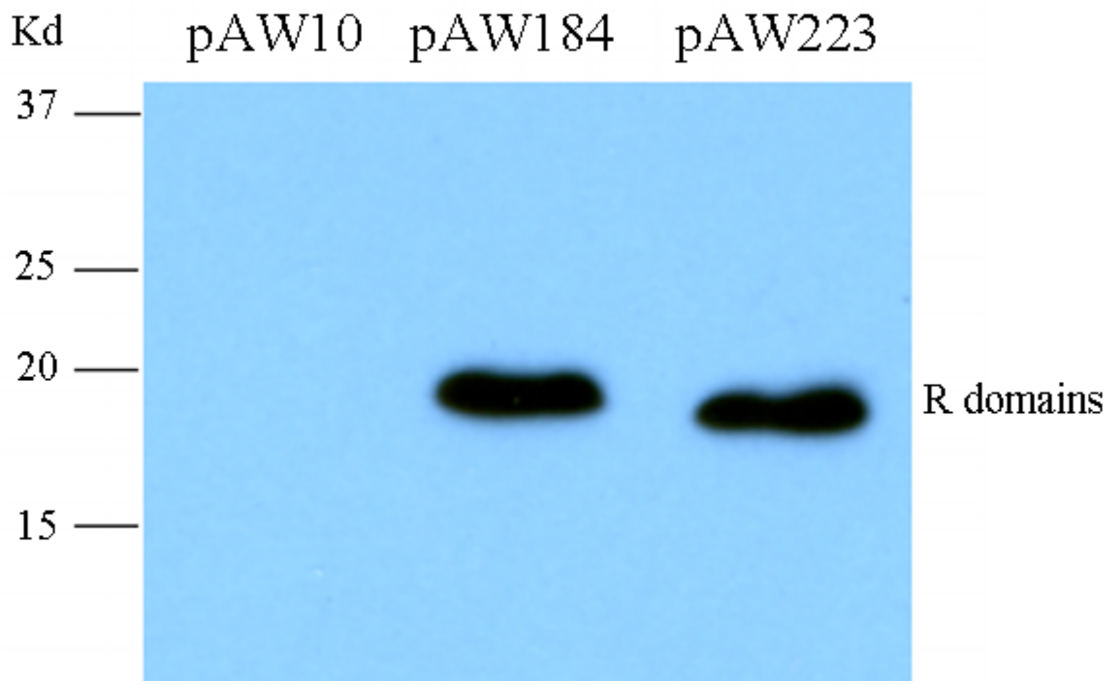

Figure S1. Immunoblot shows equivalent expression of R_A and R_{D766N} from the constitutive P_{N25} promoter in Agrobacterium. A348/pSW209Ω (carries P*virB-lacZ*) containing either pAW10 (vector), pAW184 (P_{N25}-R_A), or pAW223 (P_{N25}-R_{D766N}) were grown overnight in AB induction medium with 0.25 % glucose and 10 uM AS. Cells were concentrated to an OD600 = 10 in PAGE sample buffer and boiled 5 minutes before loading on a 15% polyacrylamide gel. Following transfer, blots were visualized with anti-RGS-6X His antibody (Qiagen),ECL anti-mouse horseradish peroxidase-linked antibody (GE Healthcare) and ECL Western Blotting Detection reagent (Amersham).

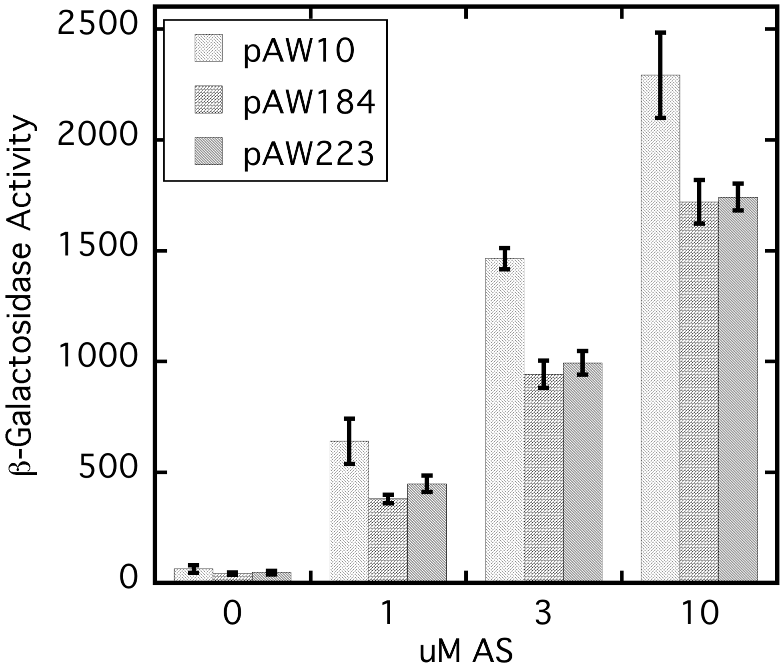

Figure S2. When A348 carries constitutively expressed *virG*, R_{D766N} appears to be as inhibitory as R_A. A348 carrying pRG109 (P_{N25}-*virG*, *virB-lacZ*) and either pAW10 (vector), pAW184 (R_A) or pAW223 (R_{D766N}) were grown in AB* induction media containing 0.22% glucose and the indicated amounts of AS.
